# Transparent Wood and Bamboo for Next-Generation Flexible Electronics: A Review

**DOI:** 10.3390/polym17141972

**Published:** 2025-07-18

**Authors:** Xiaorong Yin, Yaling Chai, Caichao Wan

**Affiliations:** College of Materials and Energy, Central South University of Forestry and Technology, Changsha 410004, China; 13077266658@163.com (X.Y.); chaiyaling2022@163.com (Y.C.)

**Keywords:** transparent wood, transparent bamboo, flexible electronics, microstructure, preparation method

## Abstract

As naturally derived composite materials, flexible transparent wood and bamboo (FTW, FTB) present notable advantages, such as straightforward preparation, high light transmittance, exceptional environmental sustainability, superior mechanical properties, low thermal conductivity, and multifunctional capabilities. Their high conductivity and sensing capabilities provide viable alternatives to conventional materials in flexible electronics. This article reviews the preparation of FTW and FTB through a top-down approach, beginning with examining how the microstructure of wood and bamboo affects the properties of these materials. It subsequently summarizes various manufacturing techniques and explores potential applications across diverse sectors. Finally, the article addresses current challenges and emphasizes the necessity for further research and innovation to promote the sustainable development of FTW and FTB in advanced applications.

## 1. Introduction

The environment and energy are essential for human advancement and existence. With the escalation of the energy crisis and heightened examination of environmental degradation, the advancement of novel energy sources and materials has become essential to meet the requirements of modern society [1,2,3]. Resource depletion from fossil fuel-related chemical and mineral overuse necessitates urgent solutions to environmental and energy challenges [4,5,6]. Wood and bamboo are the planet’s most plentiful and adaptable natural resources, providing benefits such as biodegradability, ecological sustainability, and renewability [2,7,8]. They are essential to various facets of human production and daily life. For millennia, these materials have been extensively employed in construction, furniture, and equipment [9,10,11]. However, unaltered natural wood and bamboo are limited in their high-value and functional uses due to inherent problems, such as flammability, opacity, insufficient electrical conductivity, discoloration, and decay, making them less suitable for modern human needs compared to advanced functional materials [12,13,14]. Thus, investigating innovative application areas, including flexible electronics, wearable technology, improved construction materials, and high-performance composites, augments the utility of wood and bamboo. It increases their value and effectively meets the present societal demand for sustainable resources [8,15,16].

As a burgeoning domain, flexible electronic materials present extensive developmental opportunities, with applications spanning health monitoring, wearable devices, flexible displays, and intelligent robotics [17,18,19]. This field necessitates rigorous material specifications, encompassing lightweight characteristics, great flexibility, superior optical clarity, exceptional mechanical performance, and low thermal conductivity [20,21,22]. Despite these constraints, natural wood and bamboo materials offer considerable benefits, including renewability, ecological sustainability, and biodegradability, so establishing them is highly advantageous for sustainable development [23,24]. The emergence of transparent wood and bamboo (TW, TB) materials has broadened their applications in flexible electronics [25,26]. These materials exhibit optical transparency > 90% and retain the low density and low thermal conductivity (0.2–0.4 W m^−1^ K^−1^) typical of natural wood and bamboo. After flexibility enhancement, FTW and FTB achieve fracture strengths >73 MPa and provide adjustable haze and high transparency, thereby improving light management [27,28]. Due to these advantages, FTW and FTB have become the materials of choice for critical applications in flexible electronic systems. They are widely employed in luminous electronic devices, wearable sensors, and electronic skin, effectively addressing the issues associated with the exhaustion of conventional fossil-based materials [29]. Moreover, their structure-induced macroscopic trajectories and nanopores provide these materials with significant potential for application in multifunctional smart devices [30,31,32]. Incorporating functional components by infiltration or applying surface treatments to impregnated FTW and FTB can yield functional materials with enhanced or supplementary value attributes. This substantially expands their potential applicability across diverse new domains [33,34,35].

Wood and bamboo materials that are flexible and transparent can be fabricated using either bottom-up or top-down approaches [36,37]. The bottom-up approach entails extracting nanocellulose from sources like wood by mechanical, chemical, or biological processes, subsequently pressing to fabricate films [38], and ultimately infusing these films with polymers under vacuum to produce optically transparent composites [39,40]. Although the bottom-up route yields optical transparency, its labor-intensive nature and altered microstructure limit scalability. To overcome this constraint, in a 1992 paper, Fink [41] proposed the direct transformation of entire wood into transparent wood. Recent developments have favored top-down approaches for producing optically transparent wood and bamboo composites. The method involves delignification or lignin modification of intact wood and bamboo, followed by polymer impregnation or densification [42]. In contrast to the bottom-up strategy, the top-down approach circumvents the disaggregation and reconstitution of nanocellulose, hence conserving time and resources [43]. Furthermore, it directly transforms real wood into flexible and transparent wood and bamboo materials, maintaining their intrinsic structures and imparting superior mechanical and optical qualities [44,45,46].

The progress of TW and TB has been comprehensively summarized. For example, Berglund and colleagues reported on the basic preparation and performance of TW [31], Zhu et al. reviewed the processing and application of TW and TWF [25], while Hu reported on functional applications [42] and Wu on specific construction applications [47]. However, a comprehensive review of TW and TB, primarily emphasizing their applications in flexible fields, has long been lacking. Therefore, there may be sufficient room to review flexible TW and TB from the perspective of structure–preparation–application, with a specific focus on the structure–property relationships between their microstructures and macroscopic properties, concentrating on applications of TW and TB in the field of flexible electronics.

**Figure 1:**
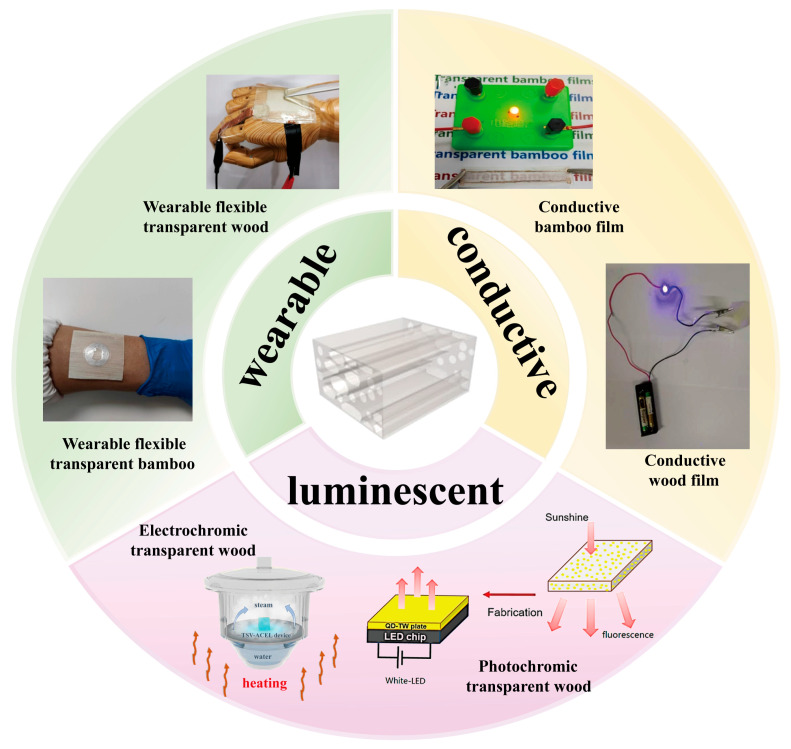
Applications of TW and TB.

This article offers a succinct overview of FTW and FTB materials produced through a top-down methodology. It begins by exploring the impact of the microstructures of wood and bamboo on the characteristics of these flexible transparent materials. Subsequently, it outlines the diverse manufacturing techniques for these materials and addresses their functionalization and applications in luminescent electronic components, flexible transparent electrodes, and sensor devices. Lastly, it evaluates recent advancements in FTW and FTB, anticipates their future functional development prospects, and delineates potential avenues for future research, as depicted in Figure 1.

## 2. Microstructure of Wood and Bamboo and Its Impact on Performance

The microstructure of wood and bamboo materials has a significant influence on their macroscopic properties [48]. FTW and FTB, produced using the “top-down” method, possess unique cell structures and pore designs that notably affect key attributes, such as strength, clarity, flexibility, and heat transfer. Consequently, a thorough investigation of the microstructures of these natural materials is essential for optimizing their use in flexible electronics.

**Figure 2:**
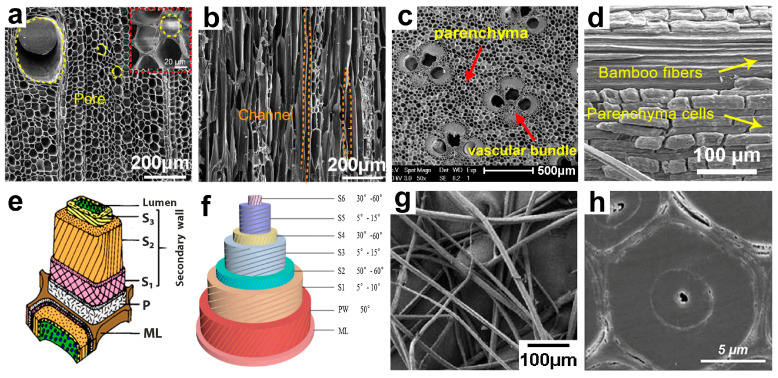
(**a**,**b**) SEM images of natural wood: (**a**) Cross-section of natural wood. (**b**) Longitudinal-section of wood. (**c**,**d**) SEM images of natural bamboo: (**c**) Cross-section of natural bamboo. (**d**) Longitudinal-section of natural bamboo. (**e**) The secondary wall of a single wood cell with three layers (S_1_, S_2_, and S_3_) surrounding the lumen. (**f**) A structure model of the Moso bamboo fiber cell wall. (**g**) Bamboo fiber. (**h**) SEM images of cross-section of single bamboo fiber ((**a**,**b**) Reprinted with permission from ref. [61]. Copyright 2021, American Association for the Advancement of Science. (**c**) Reprinted with permission from ref. [62]. Copyright 2019, The Authors. (**d**) Reprinted with permission from ref. [63]. Copyright 2022, Springer Nature. (**e**) Reprinted with permission from ref. [64]. Copyright 2017, Springer Science + Business Media B.V. (**f**) Reprinted with permission from ref. [65]. Copyright 2009, Springer Science + Business Media B.V. (**g**) Reprinted with permission from ref. [66]. Copyright 2010, Springer Science + Business Media B.V. (**h**) Reprinted with permission from ref. [60]. Copyright 2024, Elsevier B.V).

Wood and bamboo are natural organic biomass composites with diverse morphologies and variably sized hollow cells [49]. Wood predominantly comprises axially orientated tubular cells (tracheids, vessels) (Figure 2a) and radially orientated ray cells (Figure 2b) [50]. The constituent cells vary among various tree species. Wood is primarily categorized into two principal types: softwood (e.g., cedar, pine) and hardwood (e.g., balsa, oak). The fundamental distinction between these types resides in their cellular architecture [51,52]. Softwood primarily consists of tracheids in the axial direction, representing approximately 90% of its cellular composition, with diameters ranging from 30 to 50 μm. In contrast, hardwood predominantly consists of vessels, which account for 20% to 40% of its structure, with diameters between 20 and 300 μm, and wood fibers, which comprise 30% to 50%, with diameters of 10 to 40 μm. As a result, the cells in softwood are smaller and more consistently organized. Bamboo mainly has thick-walled fiber cells (60–70%), vascular bundles (which have vessels and sieve tubes), and thin-walled parenchyma tissue [53] (Figure 2c), unlike wood. Bamboo, unlike wood, does not possess transverse tissue; all cells are orientated along the axis (Figure 2d). Moreover, structural variations are evident in the cell walls, as illustrated in Figure 2e. The wood cell wall is divided into three parts: the middle lamella, primary wall, and secondary wall, based on how the biopolymers are arranged and how the microfibrils are orientated. The secondary wall consists of three layers: S_1_, S_2_, and S_3_ [54,55]. The cell wall lines up cellulose nanofibrils in parallel at specific angles (10° to 90°) to the cell axis. The angle fluctuates with the layer, with the S_2_ layer exhibiting the shortest angle, around 10° to 30°. Likewise, the bamboo cell wall possesses a multilayered architecture. In contrast to the wooden cell wall, the bamboo fiber structure has developed a highly reinforced design (Figure 2f). The S_2_ layer’s percentage rises from 85% to 95%, while the microfibril angle decreases from 5° to 10°, enabling almost axial stress transfer. Moreover, it divides into 5 to 7 alternate nanoscale microfibril lamellae, endowing bamboo with remarkable mechanical capabilities [56,57,58].

The disparities in their macroscopic mechanical properties predominantly stem from the microstructure of wood and bamboo in their natural forms. The aspect ratio of bamboo fiber cells (Figure 2g) varies from 80:1 to 200:1, markedly exceeding that of wood fibers (30:1–100:1). This aspect ratio allows bamboo fibers to convey stress more efficiently and diminish stress concentration, leading to enhanced performance of bamboo compared to wood. The axial tensile strength of bamboo is two to three times greater than that of wood [59]. Bamboo fiber cells possess small cavities and thick walls (Figure 2h) [60]. The green bamboo segment has a fiber wall-to-cavity ratio of 0.8–1.2 (with a wall thickness of 8–12 μm) and a single fiber tensile strength of 1.5–2.5 GPa, attributable to its high cellulose crystallinity (75%) and a nano-laminated architecture (consisting of 5–7 layers of microfibrils). In contrast, the yellow bamboo section has a diminished fiber wall-to-cavity ratio of 0.2–0.4 (with a wall thickness of 2–4 μm), dissipating energy via the collapse of thin-walled cells. The design of bamboo, which is strong on the outside and flexible on the inside, allows it to reach a strength of 500–600 MPa/(g/cm^3^), combining high strength with the ability to resist impacts (fracture toughness of 5–8 MPa/m^2^).

Significant variations in macroscopic qualities originate from differences in microstructure. Decoloration treatment is a critical step in the manufacture of TW and TB. This process mainly involves soaking chemicals into the tiny holes in the cell walls of wood and bamboo, which then react to remove lignin and change the natural color of the wood and bamboo to white. This technique establishes the essential prerequisite for subsequent polymer impregnation. The effective soaking of chemical agents into the tiny holes in the cell walls of wood and bamboo is important for making sure the final products are clear and see-through. The efficiency of penetration directly influences the degree of lignin removal, which, in turn, affects the transparency and haze of clear wood and bamboo. However, bamboo is notable for its high density and low porosity (15–60%), which inhibits the penetration of chemicals during treatment. The absence of transverse tissue makes it challenging to preserve bamboo’s integrity throughout processing, posing a significant barrier to FTB preparation. In contrast, wood features tubular vessels and fiber cells that create natural permeation channels. Additionally, wood exhibits a porosity ranging from 30% to 70%, facilitating the rapid infiltration of chemical reagents and the extraction of lignin. This process helps maintain the stratified porous architecture of the cellulose matrix, which is beneficial for FTW preparation. Moreover, the mechanical properties of TW and TB are essential for meeting various application requirements and quality standards [54].

In summary, bamboo demonstrates superior mechanical capabilities owing to its compact structure and considerable strength. The attributes that improve the durability and reliability of transparent bamboo also pose difficulties in chemical reagent penetration and resin impregnation, especially in the creation of flexible transparent bamboo, which may compromise its inherent mechanical capabilities. As a result, there are now limited reports on flexible transparent bamboo. Conversely, wood, which naturally contains numerous permeable channels and moderate strength, is typically more receptive to chemical treatment and resin impregnation, thereby facilitating the attainment of transparency. These properties have catalyzed research on FTW, leading to numerous applications for this material.

## 3. Preparation Method of Flexible Transparent Bamboo and Wood

**Figure 3:**
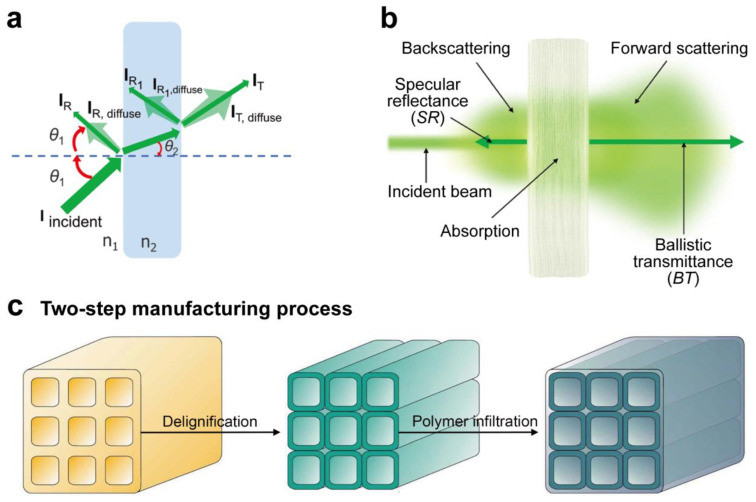
(**a**) Sketch illustration of light–solid object (RI, n_1_) interaction in a medium (RI, n_2_), where I _incident_ (incident light intensity), I_R_ (intensity of specular reflection), I_R,diffuse_ (diffusely reflected light intensity), I_T_ (ballistically transmitted light intensity), I_T,diffuse_ (diffusely transmitted light intensity), *θ*_1_ (incident angle), and *θ*_2_ (refracted angle) are presented. (**b**) Measurable photon budget components. (**c**) Two-step method for preparing TW ((**a**,**b**) Reproduced with permission from ref. [70]. Copyright 2022, Wiley-VCH. (**c**) Reproduced with permission from ref. [71]. Copyright 2020, Springer Nature Limited).

Achieving optical clarity requires systematic modulation of the native lignocellulosic framework. The primary chemical constituents of wood and bamboo are cellulose, hemicellulose, and lignin. Cellulose and hemicellulose are colorless [67], whereas lignin possesses many chromophore groups, contributing approximately 80% to 95% of the total light absorption in wood and bamboo [41,68]. The colored components induce scattering and absorption of visible light, resulting in opacity. Natural organic composite materials like wood and bamboo primarily consist of numerous tubular hollow cells with multi-layered cell walls. Numerous interfaces and pores exist between and within these wall layers and among the microfibrils. Consequently, when light illuminates the surfaces of wood and bamboo, the disparity in the refractive index between the air and moisture within the pores (mesoporous channels) and the primary constituents of the cell wall leads to the scattering, refraction, reflection, and absorption of a portion of the visible light that penetrates the interior (Figure 3a), thereby contributing to the opacity of wood and bamboo.

FTW and FTB, grounded in their chromogenic principles, can be categorized into two primary processes (Figure 3c). The initial phase entails a decolorization procedure that removes the chromophore group from lignin, resulting in delignified wood and bamboo. This procedure removes contaminants from the micropores of these materials, leading to their whitening and enabling polymer impregnation [69]. The subsequent stage involves polymer impregnation, which utilizes a transparent polymer with a refractive index akin to cellulose to rectify the disparity between the refractive indices of the cell wall and air. When light impinges against the surface of TW, the majority is transmitted, while a small fraction is scattered, resulting in TW and TB (Figure 3b).

### 3.1. Delignification Process

**Figure 4:**
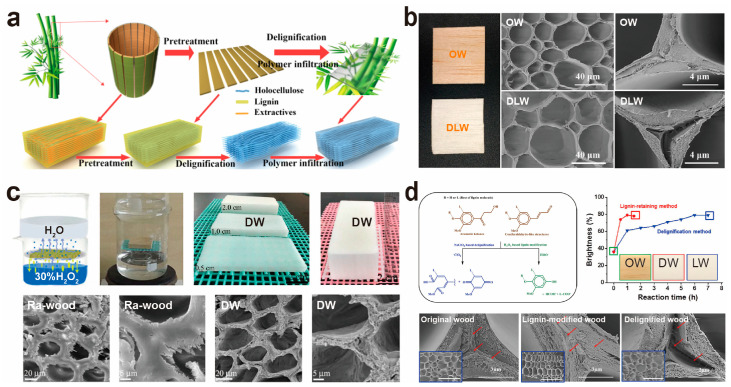
(**a**) Schematic representation of the processing route from NB to transparent bamboo. (**b**) NaClO_2_ solution delignification method: optical images and micro-structures of original wood (OW) and delignified wood (DLW). (**c**) H_2_O_2_-steam delignification method: illustrations of steam delignification, optical images of delignified wood (DW), and SEM images of Ra-wood (the lumina are perpendicular to the wood plane) and DW. (**d**) Alkaline H_2_O_2_ lignin modification method: typical reactions of NaClO_2_ solution delignification technique and alkaline H_2_O_2_ lignin modification process, as well as wood brightness before and after lignin modification and delignification, SEM images of original wood, lignin-modified wood, and delignified wood. ((**a**) Reproduced with permission from ref. [79]. Copyright 2021, American Chemical Society. (**b**) Reproduced with permission from ref. [45]. Copyright 2016, American Chemical Society. (**c**) Reprinted with permission from ref. [82]. Copyright 2019, Materials Research Society. (**d**) Reprinted with permission from ref. [85]. Copyright 2017, Wiley-VCH Verlag GmbH & Co. KGaA).

Selective elimination or chemical alteration of lignin constitutes the first critical step toward transparency. A variety of chemical treatment processes are employed, including delignification (removal of lignin) and the elimination of chromophore groups within lignin (lignin modification) [36,69,72,73,74] to achieve the decolorization of these materials. As illustrated in Figure 4a, natural bamboo undergoes either delignification or modification involving chromophore groups. The primary methods currently used for preparing TW and TB include the sodium salt method, hydrogen peroxide method, biological enzyme method, and lignin modification. However, the sodium salt method for removing lignin uses solvents and can create harmful by-products like chlorides and chlorates [75], which are bad for the environment. In comparison, the biological enzyme method and hydrogen peroxide method are considered cleaner and more environmentally friendly.

#### 3.1.1. Sodium Chlorite Process

Sodium chlorite solution is commonly used as a principal reagent in the sodium salt method for lignin removal due to its simple preparation and short reaction time. The fundamental principle is that NaClO_2_ generates unstable HClO_2_ under acidic and elevated temperatures, decomposing into Cl_2_, ClO_2_, and H_2_O. These chemicals react with the parts of lignin, like phenolic hydroxyl and carbonyl groups, breaking it down and causing it to dissolve [76,77]. Furthermore, ClO_2_ selectively oxidizes lignin while preserving cellulose integrity. Li et al. [45] subjected balsa wood (*Ochroma lagopus*) to drying at 105 ± 3 °C for 24 h and thereafter treated it with a 1.0% sodium chlorite solution, adjusted to a pH of 4.6 with acetic acid, at 80 °C to eliminate lignin until the sample achieved a white appearance (Figure 4b). This treatment markedly decreased the lignin content from 24.9% to 2.9%, while maintaining the original wood honeycomb structure and producing micrometer-scale and nanometer-scale pores (Figure 4b). Subsequent washing with deionized water or 5 mol L^−1^ H_2_O_2_ at 90 °C reduces processing time and energy consumption. Qin et al. [78] employed a two-step process to extract lignin, discovering that a minimum of 3 h of sodium chlorite treatment, followed by 1 h of hydrogen peroxide treatment, is required to remove lignin from 1 mm of balsa wood. This method effectively accelerates the removal of chlorine dioxide lignin, thereby reducing health concerns while conserving energy. The researchers adopted a similar procedure for bamboo delignification. To address the challenges associated with lignin removal from thick bamboo, Wang et al. [79] immersed natural bamboo in a 1 wt% sodium hydroxide solution prior to applying sodium chlorite for delignification. The results indicated a 60% enhancement in light transmission through the bamboo compared to untreated samples. This enhancement was attributed to the sodium hydroxide solution’s effectiveness in disrupting the linkages between lignin and hemicellulose during the pretreatment phase. The breakdown of bamboo components led to the loss of water-soluble compounds, creating microscopic breaches in the cell walls and significantly improving the ease with which substances could pass through the bamboo. These modifications facilitated the penetration of chemical agents during the subsequent lignin removal step, thereby increasing the overall effectiveness of the bamboo delignification process.

#### 3.1.2. Hydrogen Peroxide Process

The typical sodium salt delignification method produces harmful compounds during manufacture, making it unsuitable for green and ecologically friendly industrial activities. Researchers have included ecologically friendly compounds, such as H_2_O_2_, into their delignification procedures. The main idea involves the use of H_2_O_2_’s strong oxidative capacity under alkaline circumstances to selectively oxidize lignin’s functional groups, particularly phenolic hydroxyl and carbonyl groups, aiding lignin breakdown and elimination. H_2_O_2_ produces solely water and oxygen as by-products, resulting in much less wastewater creation [80]. Wu et al. [81] employed trisodium citrate dihydrate as a buffering agent for the hydrogen peroxide bleaching of basswood, successfully lowering its lignin content to 6.3% while maintaining the microstructure of the wood cells and thus producing TW. However, this method proves less efficient for thicker wood and challenging for large-area preparation. To overcome these limitations, Li et al. [82] further refined the technology, developing a steam-based method for delignifying with hydrogen peroxide. This approach exploits steam’s permeability, efficiently reducing lignin content to 0.84% and successfully fabricating large TW with a thickness of 40 mm and dimensions of 210 mm × 190 mm (Figure 4c). The steam method avoids harming the wood’s structure by not thoroughly soaking it in a large solution, which creates many tiny holes in the cell wall and corners (Figure 4c). It also markedly enhances delignification efficiency and the suitability of wood dimensions, offering fresh perspectives for the large-scale application of TW. Nevertheless, this hydrogen peroxide steam penetration method is seldom used in transparent bamboo preparation, likely due to bamboo’s high density and lack of transverse tissue, which impedes hydrogen peroxide steam penetration.

#### 3.1.3. Bio-Enzymatic Process

Like the hydrogen peroxide delignification process, biological enzyme delignification is a gentle, eco-friendly, and sustainable bleaching technique. In contrast to chemical delignification techniques, it does not depend on hazardous chemical agents, preventing the production of poisonous by-products typically associated with conventional methods. This method fundamentally relies on certain enzymes to decompose lignin, hence attaining the bleaching and translucency of wood. Laccase is extensively employed in the delignification process because it can directly utilize oxygen (O_2_) as an oxidant, facilitating the oxidation of the phenolic hydroxyl group in lignin and diminishing its degree of polymerization. Wu et al. [83] utilized the biological enzyme technique by desiccating wood samples at 100–110 °C for 24 h. Subsequently, they introduced minimal hydrogen peroxide in the presence of biological enzymes (laccase or xylanase) and modified the pH to 3–5 using glacial acetic acid. Following a reaction at 35–50 °C for 1–2 h, the samples were subjected to a treatment with a mixture of 30 wt% hydrogen peroxide and 25% ammonia water in a volume ratio of 15:1 to eliminate lignin and other chromophoric compounds, therefore achieving delignified wood. The mild conditions of the enzymatic reaction result in minimum damage to wood fibers, successfully eliminating the chromophore group while retaining fiber integrity and enhancing the mechanical qualities of wood. Enzyme activity is considerably influenced by variables such as temperature and pH level. Diverse lignin structures and compositions may necessitate particular enzyme systems for efficient degradation, constraining this approach’s universality. It has not yet been implemented in the domain of bamboo delignification. Consequently, further research must examine the enzymatic adaptability of various wood and bamboo species and lignin structures and create versatile delignification enzyme systems to facilitate the extensive application of the enzymatic approach.

#### 3.1.4. Lignin Modification Process

Lignin principally functions as a binding, supporting, and reinforcing substance in the wood and bamboo cell walls, imparting stiffness to these materials. Traditional delignification processes typically eliminate 80–90% of lignin, which may negatively impact the mechanical qualities of FTW and FTB. Thus, researchers have devised alternate lignin modification methods to attain discernible transparency while preserving or enhancing mechanical properties. Figure 4d illustrates that lignin modification is accomplished by the selective oxidation of particular functional groups within its major chromophores—o-quinone, rosin aldehyde, and aromatic ketone structures—into carboxylic acids using alkaline hydrogen peroxide. This approach preserves the essential structure of lignin while improving its chemical characteristics and color stability, negating the need for its full removal and thus maintaining the integrity of the lignin macromolecular chains. Li et al. [84] employed an alkali/hydrogen peroxide lignin modification method. The preparation procedure entailed dissolving sodium silicate (3.0 wt%), NaOH (3.0 wt%), magnesium sulphate (0.1 wt%), diethylenetriaminepenta-acetic acid (DTPA) (0.1 wt%), and H_2_O_2_ (4.0 wt%) in deionized water to formulate the lignin-modified solution. We subsequently submerged the wood matrix in this solution at 70 °C until it turned white. The findings indicated that the lignin retention technique attained a wood brightness of 79% in merely 1 h, which was one-sixth the duration necessary for NaClO_2_ solution delignification (Figure 4d). Furthermore, the alteration preserved 80 wt% of lignin. FE-SEM images revealed that the intercellular layers had only micro-scale damage post-hydrogen peroxide treatment, while notable stratification of the wood cell wall was evident after delignification with NaClO_2_ solution (Figure 4d). Xia et al. [85] employed a UV-H_2_O_2_ lignin modification system, suggesting an in situ lignin modification technique facilitated by ultraviolet radiation. Initially, natural wood underwent pretreatment with 10% NaOH, followed by the incorporation of 30% H_2_O_2_. The pretreated material was thereafter subjected to UV radiation until the wood became white. This approach preserved most of the lignin, offering a sturdy wooden framework for polymer infiltration while markedly decreasing chemical and energy usage, along with processing duration. Wang et al. [86] used a special solution with sodium silicate (3.0 wt%), NaOH (3.0 wt%), magnesium sulphate (0.1 wt%), diethylenetriaminepenta-acetic acid (DTPA) (0.1 wt%), and H_2_O_2_ (4.0 wt%) in deionized water to remove lignin from bamboo and create clear composites. These composites preserved 78% of the lignin and had a light transmittance of up to 87%, highlighting exceptional optical and mechanical characteristics.

As people pay more attention to environmental sustainability, traditional methods for removing lignin are seen as harmful because they rely heavily on chemicals and can produce toxic waste. As a result, researchers have shifted their attention to the eco-friendly production of flexible, TW and TB by employing decolorization techniques that prioritize sustainability, such as the hydrogen peroxide method and the biological enzyme approach. These methods not only reduce the utilization and release of hazardous chemicals but also provide innovative insights for the future advancement of FTW and FTB, thereby enhancing research progress and application potential in this field.

### 3.2. Polymer Impregnation Process

**Figure 5:**
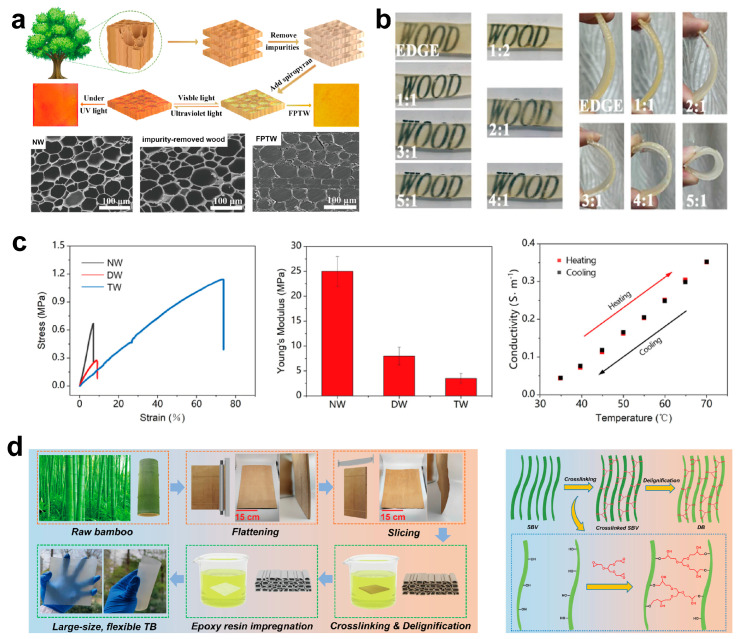
(**a**) Fabrication procedure of flexible photoresponsive TW and microstructures of NW, impurity-removed wood sample, and FPTW. (**b**) Digital photos of FTW samples prepared with different epoxy resin/EDGE ratios. (**c**) The representative stress–strain curves of NW, DW, and TW, as well as the Young’s modulus and the relationship between the conductivity of TW and temperature. (**d**) The fabrication process of large-size and flexible TB and the crosslinking and delignification processes of SBV. ((**a**) Reproduced with permission from ref. [79]. Copyright 2024, Elsevier Ltd. (**b**) Reproduced with permission from ref. [87]. Copyright 2020, Northeast Forestry University. (**c**) Reproduced with permission from ref. [23]. Copyright 2021, Elsevier B.V. (**d**) Reproduced with permission from ref. [90]. Copyright 2023, Elsevier B.V).

Post-delignification, transparent polymers are introduced to match refractive indices and reinforce the scaffold. This process entails immersing the materials in a vacuum, where external pressure effectively forces the polymer into the substrate, thereby saturating its pores and capillaries. The polymer is mixed with a curing agent in a specific ratio, after which the delignified wood template undergoes vacuum impregnation, followed by a drying phase until complete polymerization is achieved (Figure 5a). Scanning electron microscopy (SEM) images show that the original wood’s pores are mostly filled with the polymer (Figure 5a), which helps change the original wood into TW [79,87,88].

The materials used to make TW and TB usually include polymethyl methacrylate (PMMA), epoxy resin (EP), polyvinylpyrrolidone (PVP), polyvinyl alcohol (PVA), polydimethylsiloxane (PDMS), styrene, and vinyl carbazole. These polymers exhibit a refractive index akin to that of cellulose, hence diminishing light scattering and facilitating the creation of TW and TB products.

In selecting resin for FTW and FTB, achieving a balance between strength and flexibility is crucial to meet the demands of flexible electronics and wearable devices. However, relying solely on soaking these resins is insufficient to address the diverse requirements for TW and TB products. For instance, using hard resins such as EP in the preparation of flexible clear wood and bamboo materials results in products that are transparent but lack the necessary flexibility, failing to meet the fit and comfort standards for wearable devices. Conversely, opting for flexible resins like PVA may produce transparent materials that lack adequate mechanical strength, leading to reduced service life and insufficient bending resistance. Thus, to achieve the desired flexibility, it is essential to better control the mechanical response of the impregnating agents, ensuring they possess both strength and flexibility while remaining clear. Currently, there are two main ways to create impregnating agents for clear wood and bamboo materials used in flexible electronics and wearable devices: one method is to change stiff resins by adding flexibilizers to make them more flexible. Cai et al. [87] successfully synthesized TW that exhibits significant flexibility and transparency by incorporating ethylene glycol diglycidyl ether (EDGE) as an active diluent for epoxy resin. By adjusting the ratio of epoxy resin to EDGE, it is possible to create TW with varying levels of transparency and flexibility. However, as the amount of EDGE increases, both the transparency and flexibility of the FTW decrease. When the epoxy resin/EDGE ratio is set at 4/1 or 5/1, the FTW samples display remarkable transparency and exceptional flexibility (Figure 5b), meeting the criteria for diverse applications and providing a theoretical basis for expanding its use in areas such as flexible electronics. An alternative method involves incorporating plasticizers into flexible resins to enhance their mechanical strength. Subba et al. [89] utilized propylene glycol (PG) as a plasticizer in polyvinyl alcohol (PVA), creating mixed solutions of PVA-PG in various ratios. These solutions were then infused into lignin-modified wood to produce TW. The findings revealed that the light transmission of the flexible transparent bamboo improved with the addition of PG. At a PG to PVA ratio of 1:1, the optical transmittance of the resulting TW reached 80% at 550 nm. Additionally, the incorporation of PG decreased the crystallinity and glass transition temperature of PVA, thereby enhancing the flexibility and extensibility of the TW. This material demonstrated remarkable bending capabilities, enabling multidirectional flexing without breaking. Furthermore, since PVA is a biodegradable polymer, the resulting TW not only meets high-performance standards but also supports environmental sustainability, offering an innovative approach for the eco-friendly development of TW.

Alongside the previously mentioned approaches, as technology progresses and environmental consciousness increases, researchers are increasingly concentrating on creating efficient, low-consumption, and pollution-free impregnation materials to address society’s pressing need for sustainable development. Deep eutectic solvents (DES) have emerged as an optimal selection for the impregnation process owing to their low volatility, great thermal stability, exceptional biocompatibility, non-toxicity, and the absence of post-treatment necessities. Recently, DES has been employed in the fabrication of flexible TW and bamboo materials. Yang et al. [23] described a method for producing FTW using a photoinitiated polymerizable deep eutectic solvent (PDES) derived from acrylic acid and choline chloride. This study is notable as the first to utilize a polymerizable deep eutectic solvent as a filler in the production of TW via photoinitiated polymerization. The resulting TW exhibited exceptional mechanical properties, with a maximum tensile strain of 73.9% and a Young’s modulus of 3.3 MPa, which is significantly lower than that of normal wood (NW) at 24.9 MPa and dense wood (DW) at 8.1 MPa. This demonstrates the increased flexibility and deformability of the TW (Figure 5c). Additionally, incorporating choline chloride into the deep eutectic solvent enhanced the electrical conductivity and temperature-sensing characteristics of the TW. During successive heating–cooling cycles, the change in conductivity displayed a nearly linear relationship with temperature (Figure 5c), indicating remarkable stability and repeatability of the electrical signal. This underscores the material’s significant potential as a temperature sensor. Moreover, the preparation process is straightforward, time-efficient, and highly effective, establishing DES-based TW as a promising material for widespread application in future sustainable industries. This is particularly relevant in the fields of flexible electronic devices and sensors, providing unique material alternatives that integrate sustainable development with intelligent technology.

However, compared to wood, there are fewer reports of flexible transparent bamboo. Bamboo lacks transverse cellular tissue, which could explain this shortage. As a result, standard chemical treatments used for prolonged delignification of thin-layer bamboo tend to weaken transverse bonding strength, resulting in fiber bundle dissociation, structural collapse, or delamination, compromising the material’s integrity. Wang et al. [90] addressed this issue by creating scalable, large-sized, flexible, transparent bamboo (Figure 5d) using a unique “alkali pretreatment–crosslinking–delignification” approach combined with flexible epoxy resin impregnation. This method improves the bamboo pore rate and permeability through alkali pretreatment and uses 1,2,3-propanetriol diglycidyl ether (PTGE) to promote intercellular bonding (an illustration of the crosslinking and delignification processes is presented in Figure 5d). Finally, the flexible epoxy resin impregnation creates a consistent composite that greatly improves the material’s flexibility and mechanical capabilities. This technology efficiently addresses the issue of transverse dissociation in traditional bamboo processing, generating new opportunities for bamboo use in high-value-added sectors.

### 3.3. Other Emerging Preparation Methods

The vacuum impregnation method has shown certain advantages in the manufacturing of TW and TB, but it faces problems such as longer processing periods and higher polymer usage. As a result, academics are focused on inventing more efficient techniques.

**Figure 6:**
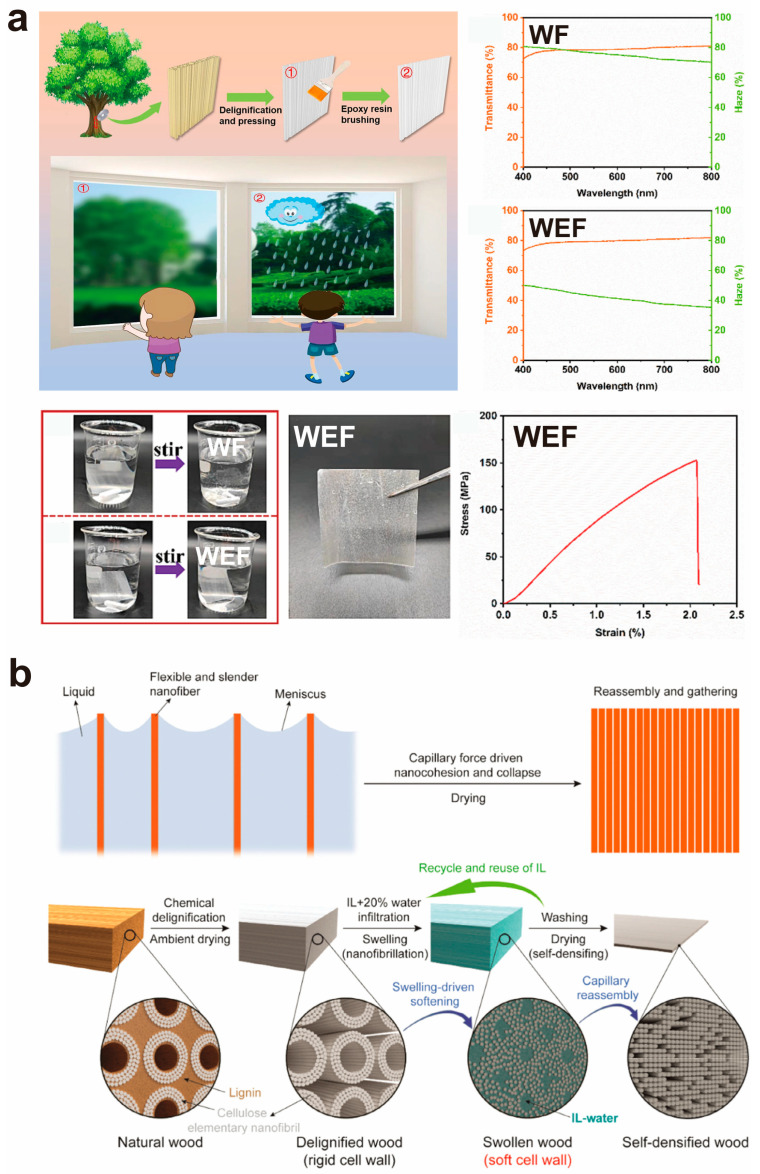
(**a**) Preparation process of transparent wood film with low haze, the optical transmittance and haze of WF and WEF, as well as the water resistance of WF and WEF. The WEF remained intact after intense stirring in water. The stress–strain curves of WEF after 30 min of water immersion along the wood fiber direction. (**b**) Schematic illustration of capillary force-induced cohesion and the gathering process of aligned flexible nanofibers upon drying, and schematic illustration of the wood cell wall structures (main part secondary layer) during the fabrication of self-densified wood, highlighting the critical role of the cell wall swelling process enabled by infiltration of the oil–water mixture solvent, which softens the cell wall and thus facilitates the reassembling of wood cell walls into self-densified wood driven by capillary force. ((**a**) Reproduced with permission from ref. [91]. Copyright 2023, Elsevier B.V. (**b**) Reproduced with permission from ref. [92]. Copyright 2024, Wiley-VCH GmbH).

Recently, Liu et al. [91] presented a simple and effective one-step brushing process that involves directly applying epoxy resin to the surface of a wood film (WF) to form wood–epoxy film (WEF) (Figure 6a). The epoxy glue on the WEF surface lowers incident light scattering, lowering haze from 76% to 43% while marginally increasing transparency (Figure 6a). Furthermore, the epoxy resin encapsulation separates the wood film (WF) from the surrounding environment, thereby preventing moisture from harming the WF. The resulting WEF has outstanding waterproof properties, retaining its integrity and strength in violently churned water (Figure 6a). This unique impregnation technology provides a new strategy for creating TW and TB, with tremendous potential for use in various sectors, particularly in wearables.

Furthermore, researchers developed a densification procedure that eliminates the need for polymer impregnation, hence minimizing the utilization of petroleum-derived products. This procedure promptly converts wood into TW composites by enhancing cellulose content, yielding an environmentally viable and sustainable alternative. For example, Chen et al. [92] demonstrated a self-densification method utilizing capillary forces. Subsequent to delignification, the modified wood is subjected to expansion using a solution of ionic liquid (1-ethyl-3-methylimidazolium acetate, [EMIM][OAc]) and water to facilitate cell wall softening. In the ensuing drying process, the wood cells inherently collapse due to capillary forces, creating a layered structure that leads to self-densification and the effective fabrication of lightweight, high-strength, and transparent wood films (Figure 6b). This self-densification relies on the expansion action induced by the ionic liquid–water mixture within the wood cell wall, while the evaporation of moisture, driven by capillary forces during drying, causes the collapse of the wood cell wall, thereby obviating the necessity for conventional thermal pressing. This significantly decreases energy consumption and obviates the necessity for polymers, leading to effective wood conversion and performance improvement.

In summary, polymer impregnation is the primary technology for producing FTW and FTB. Frequently utilized polymers encompass PMMA, EP, PVP, and PVA, which possess a refractive index similar to that of cellulose, thereby diminishing light scattering and enhancing transparency. Various polymers substantially influence the performance of FTWs and FTBs; for instance, PVA augments flexibility, whereas EP predominantly enhances mechanical strength. Nonetheless, conventional methods depend on petroleum-based feedstocks, leading to environmental pollution and cost concerns. Researchers have recently devised more environmentally sustainable and efficient preparation methods, including one-step brush-coating and self-compacting technology. These innovations not only streamline the process and enhance the waterproof performance of the materials but also diminish dependence on the conventional hot-pressing technique. These novel methods markedly diminish environmental impact and, in certain instances, enhance material performance. Nevertheless, these innovative techniques impose stricter requirements on processing conditions, including the need for meticulous regulation of ambient humidity and temperature and the material’s pretreatment process, which may otherwise result in issues such as inconsistent impregnation or residual air bubbles.

In conclusion, conventional polymer impregnation methods remain competitive in large-scale manufacturing due to their established nature and extensive applicability. Conversely, novel preparation techniques exhibit significant potential in particular application contexts and environmental conservation, offering a new pathway for the sustainable advancement of TW and TB.

## 4. Application of TW and TB

With the increasing demand for electronic devices such as laptops and smartphones, traditional substrate materials such as glass and plastic, which are non-biodegradable and non-renewable, have become more environmentally problematic. The extensive usage of fossil-based plastics has led to the inclusion of dangerous compounds in electronic waste, such as gallium arsenide and heavy metals, which pose substantial hazards to both the environment and human health. As a result, finding an alternative material that provides high transparency, flexibility, printability, and environmental friendliness has become critical. FTW and FTB, as new sustainable materials, have significant application possibilities. These materials also have great optical qualities, with light transmission of up to 90%, as well as excellent mechanical properties. In the field of flexible electronic devices, FTW and FTB can replace standard plastic substrates in the fabrication of flexible screens, sensors, and wearable gadgets, reducing the environmental impact of electronic waste. Their intrinsic flexibility and plasticity provide additional opportunities for personalized design, improving their use in flexible electronic devices. Because of their remarkable environmental properties and versatility, FTW and FTB are poised to find extensive application in optoelectronic devices and environmental materials, providing creative answers to traditional materials’ environmental concerns.

### 4.1. Flexible Transparent Wood

FTW recognized as a biodegradable and eco-friendly material, exhibits significant transparency, adequate mechanical strength, and exceptional thermal stability. Additionally, it offers substantial prospects for additional functionalization via composite and coating techniques. Incorporating functional chemicals into resin solutions or wood templates enables the production of multifunctional TW, significantly improving its performance and expanding its applicability across diverse industries.

#### 4.1.1. Light-Emitting Electronic Components

**Figure 7:**
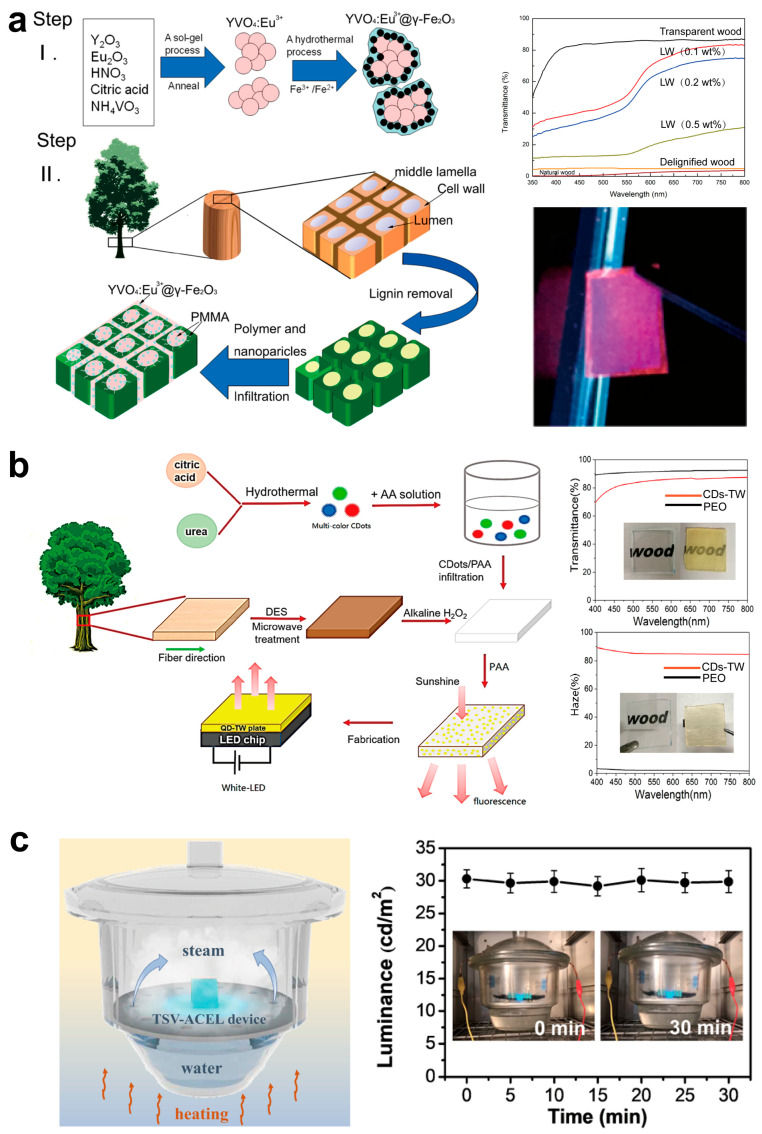
(**a**) Experimental strategy for the fabrication of luminescent wood, the transmittance of wood samples obtained from different processing stages, and luminescent wood with γ-Fe_2_O_3_@YVO_4_: Eu^3+^ NP concentration of 0.1 wt% under UV excitation at 254 nm. (**b**) Fabrication strategy of a white LED based on a UV light-emitting diode chip with multiple-color-emission CDs-TW as the encapsulating materials and optical transmittance and haze of PEO and CDs-TW with a thickness of 550 μm. (**c**) Schematic of an alternating current electroluminescent (ACEL) device with a flexible transparent wood film as a substrate, as well as the brightness variation of the TSV-ACEL device at high temperature (90 °C) and relative humidity (>90%, 30 min) (220 V, 1 kHz). ((**a**) Reprinted with permission from ref. [93]. Copyright 2017, American Chemical Society. (**b**) Reprinted with permission from ref. [94]. Copyright 2018, American Chemical Society. (**c**) Reprinted with permission from ref. [95]. Copyright 2019, American Chemical Society).

In luminous materials research, lanthanide ion-doped nanoparticles have attracted considerable interest due to their distinctive chemical characteristics. These nanomaterials demonstrate exceptional properties like scintillation, low toxicity, resistance to photochemical degradation, and photobleaching. Embedding luminous nanoparticles in wooden templates facilitates the creation of luminescent wood composites with enhanced characteristics, hence broadening the applicability of wood materials in the optical domain. In this respect, Gan et al. [93] initially integrated magnetic, luminous γ-Fe_2_O_3_@YVO_4_: Eu^3+^ nanoparticles with PMMA, which had a corresponding refractive index, to effectively create a unique magnetic luminescent TW composite material (Figure 7a). The resultant wood composite, incorporating 0.1 wt% γ-Fe_2_O_3_@YVO_4_: Eu^3+^ nanoparticles, demonstrated a high transmittance of 80.6% across a broad wavelength spectrum of 350–800 nm (Figure 7a) and displayed a vivid photoluminescent hue under UV stimulation at 254 nm (Figure 7a). The resin occupying the cellular cavities improved the contact among cellulose nanofibers, thus imparting exceptional thermal stability to the wood composite. Thermogravimetric analysis (TG) results demonstrated that the composite material experiences mass loss only owing to the degradation of the wood component and PMMA within the temperature range of 260–420 °C. Compared to the DTA results of PMMA and natural wood, the exothermic peak of LW was diminished and displaced to elevated temperatures, signifying enhanced thermal stability. This attribute indicates that the wood composite can preserve structural integrity at temperatures exceeding 260 °C, showcasing a notable thermal stability advantage compared to real wood. Moreover, Gan et al. performed water immersion tests on wood composite materials. The findings revealed that the treated luminescent wood (LW) displayed markedly reduced volumetric changes in water relative to natural wood. Specifically, after 60 days of immersion, the volume of natural wood increased by 40.4%, while the luminescent wood (LW) exhibited only a 15.8% increase, thereby illustrating its enhanced dimensional stability and further substantiating the benefits of this composite material regarding environmental stability. In addition, the incorporation of γ-Fe_2_O_3_ conferred superparamagnetism to TW. These attributes render TW exceptionally promising for applications in green LED lighting, luminous magnetic switches, and anti-counterfeiting technologies. Furthermore, due to the superior optical, mechanical, and thermal characteristics of FTW, FTW has emerged as an optimal alternative to epoxy resin for the primary packaging material of light-emitting diodes (LEDs). Bi et al. [94] created multicolor luminous, carbon dots/transparent wood (CDs-TW) by amalgamating three distinct color quantum dots and subsequently integrating the fluorescent quantum dots into delignified wood using in situ polymerization (Figure 7b). CDs-TW demonstrates an optical transmittance of up to 85% and, in contrast to pure epoxy resin (PEO), presents a significant haze of 85% throughout the visible light spectrum (Figure 7b). The elevated transmittance and haze characteristics of CDs-TW make it promising for light management applications. Moreover, its longitudinal mechanical strength is improved to 60.92 MPa, much exceeding that of natural wood. Moreover, when utilized as an encapsulation film for white LEDs, the transparent wood film exhibits exceptional color properties, providing a sustainable, uncomplicated, and energy-efficient approach to manufacturing metal-free wood-based W-LED encapsulation materials. A separate study utilized transparent wood film as the substrate for alternating current electroluminescent (ACEL) devices, successfully developing a transparent wood ACEL device that demonstrated a brightness of 18.36 cd/m^2^ (with a voltage of 220 V and a frequency of 1 kHz) (Figure 7c). After undergoing 500 bending test cycles, the thin-layer resistance showed stability, ensuring consistent conductivity. The transparent wood film–alternating current electroluminescent (TWF-ACEL) device can endure a 90 °C environment with 90% humidity for up to 30 min, during which its luminous intensity decreased from 30.31 cd/m^2^ to 29.16 cd/m^2^ (Figure 7c). This performance is comparable to traditional plastics such as PET, nylon, polyethene, polyvinyl chloride, and epoxy resin, offering a novel alternative for display and lighting technologies in extreme high-temperature and humidity conditions [95].

#### 4.1.2. Flexible Transparent Electrodes

**Figure 8:**
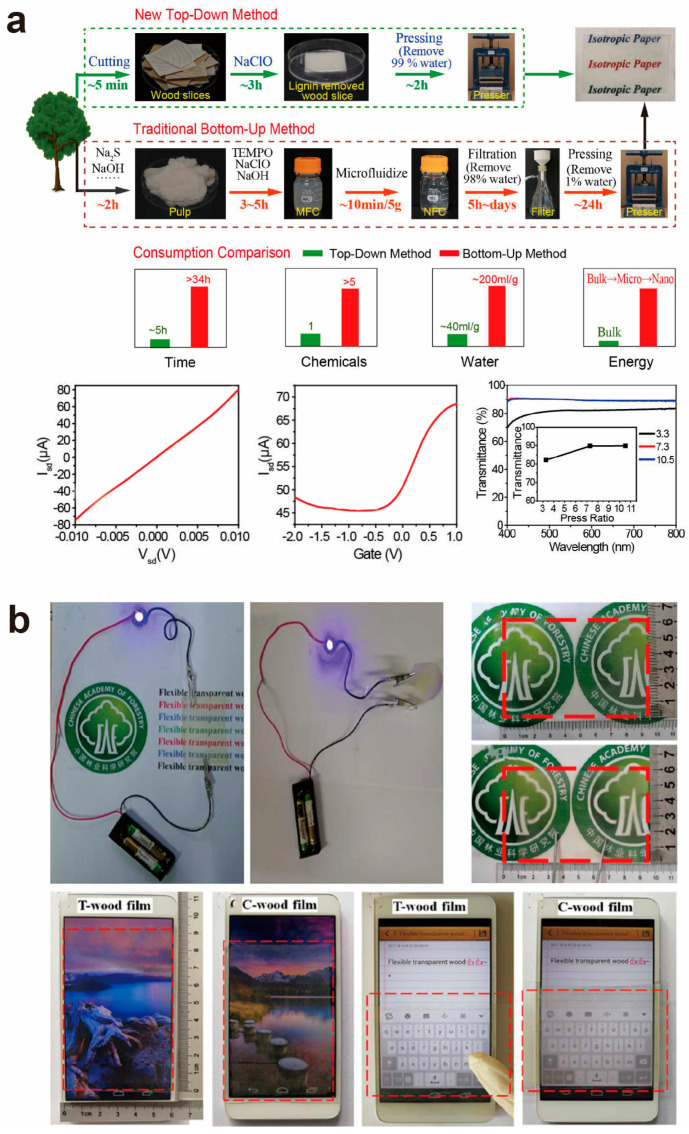
(**a**) Comparison of top-down versus bottom-up methods for making isotropic transparent paper in terms of production process, time, estimated consumption of chemicals, water, and energy. (**b**) Light-emitting diodes were lit up with T-wood film/341 mg/m^2^ silver nanowires in natural and bent states, images showing light transmittance and haze of the wood film, and photographs of a flexible transparent wood film covered with a moving mobile phone display, respectively. ((**a**) Reprinted with permission from ref. [96]. Copyright 2018, American Chemical Society. (**b**) Reprinted with permission from ref. [97]. Copyright 2018, The Royal Society of Chemistry).

Traditionally, electrodes for flexible electronic devices are primarily composed of fossil-derived polymers; however, the non-biodegradable nature of these materials has resulted in significant environmental challenges. To address this, FTW exhibiting excellent transparency and a low thermal expansion coefficient has been investigated as a substitute for conventional substrates. The fabrication of conventional flexible transparent wood films often employs a “bottom-up” methodology, necessitating intricate fiber extraction and purification processes to isolate cellulose nanofibers from wood and construct the films. This technology may produce high-quality transparent films, but its complexity and expense impede general adoption. To this end, Zhu et al. [96] introduced a “top-down” methodology for directly fabricating isotropic transparent films from anisotropic wood. In contrast to conventional techniques, this novel technology swiftly, economically, and sustainably generates high-transparency, high-haze, flexible, and biodegradable transparent paper via straightforward delignification and flattening processes (Figure 8a). The researchers effectively manufactured graphene transistors on these TW substrates. The graphene transistors demonstrate standard ohmic contact properties, characterized by a linear correlation between the source–drain current and the source–drain voltage (Figure 8a), signifying minimal contact resistance between the graphene and the electrodes, thereby facilitating efficient current conduction through the graphene sheets. Moreover, via electrolyte gating, graphene transistors have bipolar characteristics, with the source–drain current fluctuating in relation to the gate voltage. This facilitates effective conductivity regulation at low voltages (−2 V to 1 V), as illustrated in Figure 8a. This property is very vital for low-power electrical systems and further highlights their remarkable conductivity. Furthermore, the transparent wood film exhibits a tensile strength of around 150 MPa and shows no notable cracks or fractures in various bending and folding tests, underscoring its remarkable flexibility. These results demonstrate that the transparent film serves as an optimal substrate for disposable electrical and optical devices. Furthermore, by modifying the pressing ratio, the film’s transmittance may be adeptly customized; an increase in the pressing ratio corresponds with an enhancement in overall transmittance, as demonstrated in Figure 8a. This versatility enables the transparent film to accommodate diverse application conditions. Tang et al. [97] improved the surface of the transparent wood film by depositing silver nanowires (AgNWs), yielding a composite film that exhibits higher conductivity and transparency, as illustrated in Figure 8b. Its conductive performance competes with that of conventional transparent conducting oxides (ITO), while offering reduced cost and improved flexibility. This novel transparent wood film is appropriate for electronic devices, including solar cells and flexible displays, and demonstrates potential for practical uses such as screen protectors. When designs are inscribed on a smartphone screen using a finger, it effectively exhibits the text “flexible transparent wood” (as illustrated in Figure 8b). Moreover, it possesses superior anti-glare characteristics for outdoor displays.

#### 4.1.3. Flexible Sensor Components

**Figure 9:**
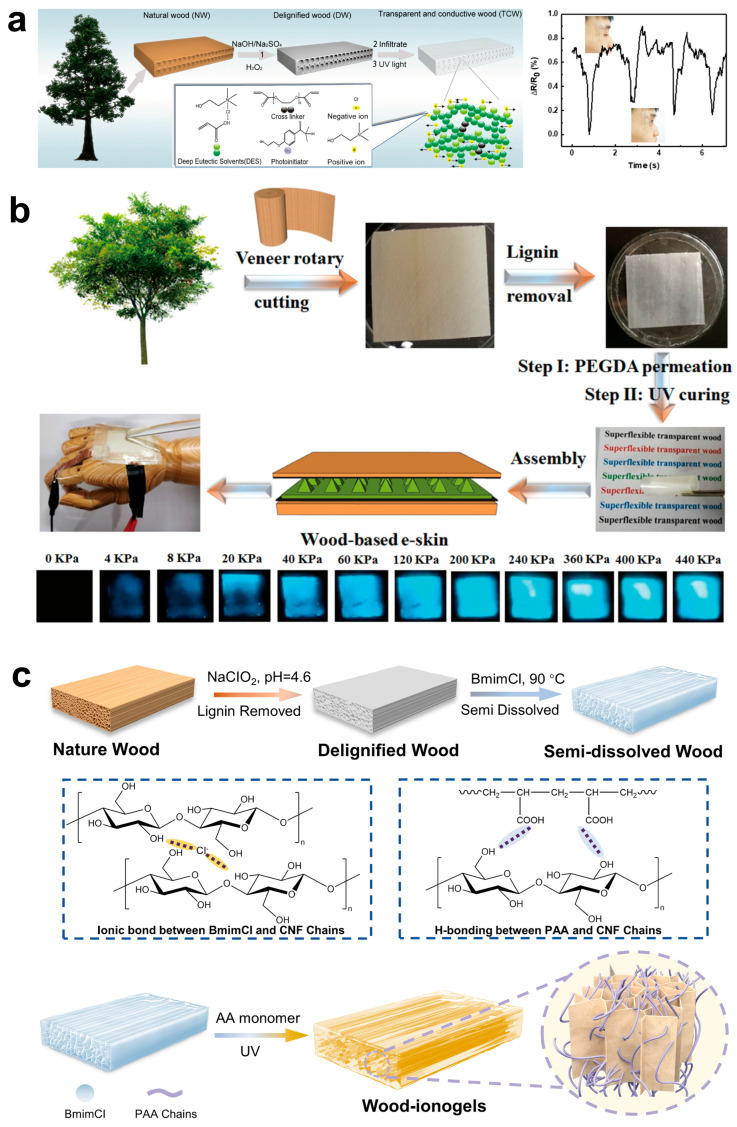
(**a**) The schematic illustrates the fabrication of TCW composites through three steps: (1) delignification, (2) permeation, and (3) photopolymerization, as well as the use of TCW as a strain/touch sensor for monitoring human blinking. (**b**) Instructions for making a wooden e-skin and images of the transient luminescent response of the e-skin with increasing pressure. (**c**) Design schematic illustration of SDW-PAA ionogels: Preparation process of semi-dissolved wood and AA impregnation into semi-dissolved wood for the preparation of SDW-PAA ionogels. ((**a**) Reprinted with permission from ref. [21]. Copyright 2019, American Chemical Society. (**b**) Reprinted with permission from ref. [18]. Copyright 2021, Elsevier B.V. (**c**) Reprinted with permission from ref. [98]. Copyright 2024, Elsevier B.V).

The demand for flexible sensor devices in sectors such as wearable technology, intelligent robotics, and health monitoring is increasing quickly. Flexible sensors translate pressure, temperature, and strain into electrical signals while conforming to complex motions and environments. Nevertheless, conventional flexible sensor devices mainly depend on petroleum-derived polymers for their substrates and electrodes, which creates constraints due to the non-renewable nature of these materials. As a natural material, FTW exhibits exceptional flexibility and stretchability while possessing excellent optical transparency and conductivity. These attributes render it an optimal substitute for conventional materials in flexible sensor applications. Wang et al. [21] documented a unique stretchable and conductive wood-based composite synthesized using in situ photopolymerization of a deep eutectic solvent (PDES) (Figure 9a). Due to the preceding hydrogen-bonding interactions between PDES and BWS, the resulting transparent conductive wood (TCW) attains a tensile strain of up to 80% and an electrical conductivity of 0.16 S/m. Notably, when the TCW material was affixed to human skin to observe subtle movements caused by micro-expressions, the findings indicated that the sensor swiftly alternated between the loading and unloading phases. The signal response was consistently reproducible and stable for matching motions, displaying notable variations in shape and amplitude across different activities (Figure 9a). These attributes underscore the considerable potential of TCW as a high-sensitivity sensor. Yang et al. [23] subsequently reported using the same PDES to fabricate FTW, putting the resultant material to thermal cycling tests. As the temperature climbed from 30 °C to 70 °C, the electrical conductivity of TW exhibited a monotonic rise and displayed consistent conductivity variations throughout 10 heating–cooling cycles, with little significant noise. This exceptional temperature sensing capability establishes TW as a viable material for temperature sensor applications. Moreover, the functional alteration of FTW markedly improves its sensing capabilities. Tang et al. [18] used AgNW on the fabricated ultra-flexible transparent wood to develop a TW-based electrode for a pressure sensor. They incorporated a stimulus-responsive layer with pyramid microstructures between two TW-based electrodes, creating a flexible wooden electronic skin that demonstrates dual responsiveness in capacitance and optics (Figure 9b). This skin is perceptible to both the unaided eye and tools. The wood-based electronic skin produces electroluminescent responses within the human pain pressure range (0 to 150 kPa) while preserving great sensitivity (>0.15 kPa^−1^). Figure 9b distinctly depicts luminescence intensity fluctuating from 0 to 440 kPa, emphasizing its use in wearable electronics, artificial intelligence, and soft robotics. Moreover, the substrate material of the electronic skin—super-flexible transparent wood film (STW)—exhibited remarkable thermal stability. It preserved its shape and transparency for 30 min at temperatures of 100 °C, 130 °C, and 150 °C, demonstrating commendable thermal cycling stability and adaptability to varying thermal environments. Recent reports indicate that anisotropic ionogels (SDW-PAA ionogels) are synthesized by infusing acrylic acid (AA) monomers into delignified wood. This technique utilizes ionic solutions to partially dissolve delignified wood, resulting in a very porous wood structure. Acrylic acid (AA) monomers are subsequently introduced into this framework, and polymerization is initiated by ultraviolet light, yielding ionogels with a double-network architecture (Figure 9c). This distinctive design maintains the wood’s inherent anisotropy, highlighting remarkable flexibility, conductivity, swift responsiveness, and extraordinary mechanical qualities, including a tensile strength of 9.0 MPa in the longitudinal direction. Additionally, this ionogel exhibits remarkable environmental stability, maintaining its integrity at room temperature for over 30 days. Across a temperature spectrum of −20 °C to 100 °C, its storage modulus consistently exceeds the loss modulus, indicating effective anti-drying properties and thermal cycling resilience. These attributes render FTW highly suitable for extensive applications in areas such as electronic skin and wearable medical devices. [98]

The utilization of TW and TB in flexible electronics is progressively broadening, primarily targeting compact devices and encompassing sectors such as wearable technology, intelligent robotics, and sensor substrate materials for health monitoring. These sectors are deemed more viable for practical implementation due to their substantial demand for material flexibility, biocompatibility, and eco-friendliness. However, several challenges persist, including material compatibility concerns, energy consumption in production, cost management, and the maintenance of consistent device performance during mass production. The challenges arise partly from wood’s porous composition and intrinsic anisotropy, which can influence the stability and longevity of electronic devices. Furthermore, the biodegradability of wood, although environmentally beneficial, must be evaluated for long-term stability. To mitigate these issues, TW and TB can be refined from current frameworks, their environmental stability can be augmented through advanced functionalization treatments, and manufacturing process parameters can be optimized to minimize costs and guarantee performance consistency.

### 4.2. Flexible Transparent Bamboo

Bamboo predominantly consists of vascular bundles and thin-walled cells, and current research on flexible transparent bamboo is quite scarce.

**Figure 10:**
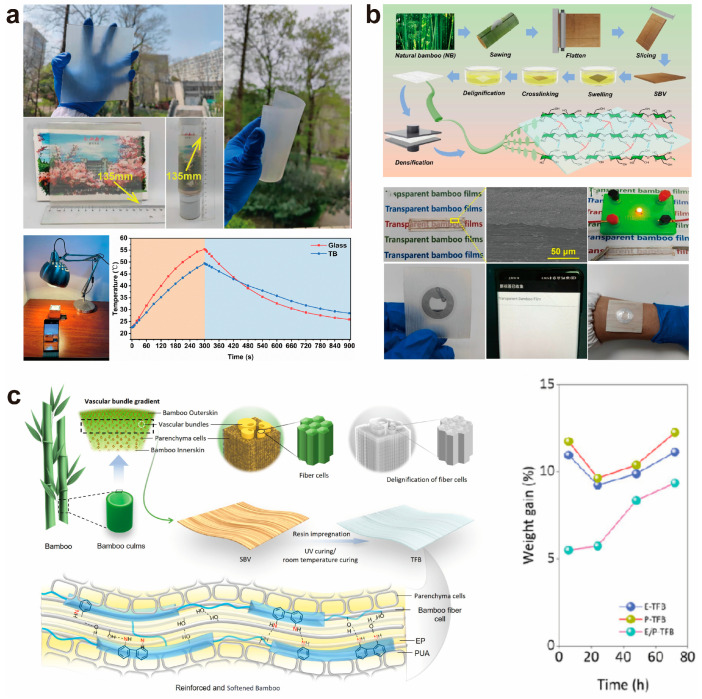
(**a**) A large-size, flexible TB with dimensions of about 135 mm × 135 mm, an exterior display, an image of a home-made experimental model house (a foam square box wrapped in aluminum foil and covered with glass or TB on one side), and continuous simulations of temperature and time changes of TB and glass under solar radiation and shutdown conditions. (**b**) The preparation process of transparent bamboo films and the application of BF as a substrate for optoelectronic devices. (**c**) Schematic representation of the processing route from NB to TFB and weight gain rate curve of E-TFB, P-TFB ((**a**) Reprinted with permission from ref. [90]. Copyright 2023, Elsevier B.V. (**b**) Reprinted with permission from ref. [99]. Copyright 2024, Elsevier B.V. (**c**) Reprinted with permission from ref. [100]. Copyright 2025, Elsevier B.V).

In the field of energy-saving buildings, Wang et al. [90] utilized sliced bamboo veneer (SBV) as the substrate. This substrate underwent preprocessing with sodium hydroxide, PTGE cross-linking, and NaClO_2_ delignification, followed by impregnation with flexible epoxy resin, successfully fabricating an expandable, large-sized (135 mm × 135 mm) flexible transparent bamboo (Figure 10a). At a thickness of 1 mm, this material exhibited 80% transparency and 72% haze, with a tensile strength of 78.5 MPa, significantly surpassing all previously reported flexible wood (TW). Additionally, the researchers used a self-made aluminum foil-wrapped foam cubic model house, with one side covered by glass and the other by TB windows. Simulating sunlight exposure revealed that the glass-windowed house heated up faster, rising from 22.6 °C to 55.4 °C within 300 s, while the TB-windowed house only reached 49.4 °C. After turning off the light source, the glass-windowed house cooled down more rapidly, dropping from 55.4 °C to 25.9 °C, whereas the TB-windowed house decreased from 49.4 °C to 28.5 °C (Figure 10a). This demonstrates that TB windows offer excellent thermal insulation and indoor temperature regulation, making them a promising novel transparent window material for energy-saving buildings.

Liu et al. [99] have developed high-performance flexible transparent bamboo for optoelectronic devices through alkaline pretreatment, crosslinking, delignification, and compression densification (Figure 10b). This material, noted for its remarkable transparency, strength, toughness, and flexibility, shows significant promise as a substrate for optoelectronic applications. In experiments, conductive ink was uniformly applied to the bamboo film (BF) surface, achieving strong adhesion and facilitating circuit assembly and diode illumination (Figure 10b), indicating its potential to substitute for petroleum-based plastic films as a conductive substrate. Additionally, NFC circuits based on BF can be quickly read by devices. They can adhere to the skin, functioning as wearable technology (Figure 10b), thus presenting an innovative avenue for electronic skin development.

A recent study detailed the creation of a flexible transparent bamboo-based composite (TFB) utilizing a dual-resin mixing approach, demonstrating exceptional mechanical and optical properties. The bamboo material underwent delignification to yield a bamboo fiber framework, which was subsequently infused with a resin mixture of epoxy (EP) and polyurethane acrylate (PUA) through vacuum-assisted impregnation. A two-step curing process was implemented, incorporating ultraviolet light and natural curing (Figure 10c). The resultant TFB exhibited a tensile strength of 169.03 MPa, a light transmittance of 81.47%, and an optical haze of 72.43%, significantly surpassing natural bamboo and other bamboo-based composites. The integration of PUA augmented the material’s flexibility and substantially enhanced its resistance to yellowing. Additionally, TFB displayed a low thermal conductivity of 0.20 W/m·K and exceptional water stability, with a weight increase rate of less than 10% over 72 h (as illustrated in Figure 10c). It also maintained consistent performance when subjected to 90 °C and 90% relative humidity for 30 min. These characteristics render TFB highly advantageous for applications in energy-efficient building materials, wearable technology, and sustainable packaging [100]

In summary, bamboo’s exceptional strength, toughness, low thermal conductivity, rapid growth, and sustainability render it an optimal raw material for the fabrication of FTBs. FTBs exhibit remarkable optical and mechanical properties, including high transparency, flexibility, and low thermal conductivity, which are attributable not only to the inherent qualities of the bamboo but also to the sophisticated preparation process employed. These attributes render FTB highly promising for application in flexible electronic devices. With ongoing advancements in preparation technology, FTB is anticipated to find extensive use across various sectors, including wearable devices, sustainable packaging, and smart buildings, thereby facilitating its industrialization. Simultaneously, using bamboo’s distinctive cellular architecture and chemical properties to create novel applications, including advanced electronic skin and health monitoring devices, would yield revolutionary solutions for the sustainable advancement of flexible electronics technology.

Excellent mechanical strength, optical qualities, and environmental sustainability make FTW and FTB viable candidates for next-generation flexible electronic devices. They are also comparatively more straightforward and less expensive to prepare than biomass-derived substrates made using bottom-up techniques, like silk protein films (SPF), bacterial cellulose films (BCF), and cellulose nanofibers (CNF). In the case of CNF, for example, its preparation typically involves subjecting a suspension of cellulose nanofibers to oxidation. This process requires precise control of the concentration of oxidant and reaction time in order to ensure the structural integrity and performance of cellulose nanofibers. In contrast, FTW and FTB are typically prepared using a top-down approach through delignification and polymer impregnation. In addition to avoiding harrowing chemical extraction and purification processes, this method better maintains the natural structure of bamboo and wood to produce superior mechanical qualities, high optical transmittance, and precise haze control by modifying the material’s microstructure, offering a broad range of applicability for various application scenarios. For instance, they can be used as substrates for flexible displays and sensors in the electronics field, offering excellent optical properties and mechanical flexibility. FTW and FTB have great potential for multifunctional integration, and their properties can be further enhanced by lamination and coating techniques to extend their application range in various industries.

In summary, FTW and FTB exhibit significant advantages in terms of preparation simplicity, cost-effectiveness, and optical performance compared to other biomass-derived substrates, such as CNF, BCF, and SPF. Specific comparisons are listed in Table 1.

## 5. Critical Areas, Summary, and Outlook

As promising composite materials, FTW and FTB present numerous advantages, such as lightweight, adjustable haze, superior light transmission, substantial mechanical toughness, and high thermal stability. Additionally, research into their functionalization indicates significant potential for applications in electronic skin, sensor devices, light-emitting electronic components, and energy storage materials. This article begins by analyzing how the microstructure of wood and bamboo affects the properties of FTW and FTB. It subsequently details the preparation methods for these materials and concludes with an overview of their potential applications in light-emitting electronic components, flexible transparent electrodes, and flexible sensor devices.

The investigation into the advancement and utilization of flexible, transparent wood and bamboo-derived materials, despite their distinct advantages, faces several urgent challenges that necessitate prompt resolution. These challenges predominantly pertain to the following critical areas:

1. Developing FTW and FTB materials requires more environmentally sustainable delignification methods and lignin-preserving decolorization techniques. Conventional delignification processes utilize excessive chemical reagents and cause considerable environmental pollution. While some research has proposed greener delignification approaches, the resultant wood and bamboo still necessitate significant organic solvents to eliminate residual chemicals. Consequently, forthcoming research may enhance the sustainable development of these materials by recycling chemical waste or substituting current reagents with eco-friendly alternatives. Furthermore, lignin is essential for wood and bamboo’s adhesion and structural integrity. The delignification process frequently disrupts their inherent layered architecture, undermining mechanical properties. This challenge can be mitigated by judiciously modifying lignin while preserving its supportive role and implementing comprehensive material alterations. Future investigations may also examine lignin recycling, reinserting extracted lignin into the voids of wood and bamboo, optimizing raw material utilization for sustainable production and achieving a closed-loop production cycle.
2. FTW and FTB materials possess considerable potential for functionalization. However, existing research predominantly focuses on augmenting singular functions, with limited investigations into multi-functional integration. This considerably constrains their applicability in flexible electronics and wearable devices. Future research should emphasize functional integration to create FTW and FTB materials with varied functionalities. Nonetheless, during the functionalization process, it is essential to mitigate interference among different functional components and to prevent detrimental effects on the material’s flexibility and transparency. For example, in flexible electronics, TW and TB are promising as wearable sensors. However, their comprehensive properties, such as conductivity, mechanical stability, and transparency, require meticulous assessment before practical implementation. The requirements of wearable devices can be adequately fulfilled by attaining high conductivity and substantial mechanical stability through functional modifications while maintaining material transparency. Thus, the advancement of multifunctional TW becomes a crucial research focus for the future.
3. Future research may concentrate on broadening the utilization of flexible and TW and TB materials in optoelectronic devices and wearable technologies. This includes the advancement of flexible screens with integrated light-emitting and display capabilities and wearable devices for health monitoring, such as real-time tracking of heart rate, body temperature, and other physiological signals. At the same time, the materials’ durability and environmental adaptability must be enhanced to address the diverse challenges presented by complex environments, ensuring long-term stability and reliability. It may also investigate its use in domains such as intelligent apparel and environmental monitoring sensors to advance the evolution of associated technologies further. These applications involve physical contact, mechanical friction, and intricate operating environments. Extended use can lead to surface degradation and internal structural damage, especially in contexts characterized by human movement, temperature variations, and humidity fluctuations. Consequently, it is imperative to enhance these materials’ durability, mechanical stability, and environmental adaptability to ensure their reliability and longevity in practical applications. Moreover, given the prospective uses of TW and TB in flexible electronics, their environmental stability is crucial. It is essential to systematically assess the performance of these materials under diverse environmental conditions, including humidity, temperature fluctuations, and ultraviolet exposure. Enhancing their environmental stability through the incorporation of functional components or the application of functional coatings could broaden their applicability in intricate environments. Future research should prioritize these properties and examine the performance of FTW and FTB under complex conditions to fulfil the requirements of diverse applications.
4. The dimensions of TW and TB predominantly range from 1 to 10 cm. Although some research has achieved advancements in producing larger sizes, the current dimensions remain inadequate for the stringent requirements of various emerging sectors, such as flexible electronic displays. The developmental trajectory in these areas necessitates larger TW and TB materials to accommodate the demands of large-scale, integrated devices and to enhance display quality. However, as size and thickness increase, the efficacy of delignification and polymer impregnation diminishes, adversely affecting the materials’ essential optical and mechanical properties. To mitigate this challenge, integrating photocatalytic oxidative modification with multilayer TW processing technology offers a partial remedy. In addition, the variability in raw material sources and microstructural heterogeneity can influence product quality stability. To address these issues, sustainable sourcing systems may be implemented to guarantee a reliable supply of high-quality raw materials. Continuous delignification processes can be refined to enhance productivity and stability. Roll-to-roll technology can implement functional treatments to enhance material durability and environmental resilience. Furthermore, standardized testing methodologies and quality control systems can guarantee consistent performance across batches. Through these integrated strategies, the mass production of flexible and transparent wood and bamboo materials can be significantly advanced to facilitate their extensive application in optoelectronic devices and wearable technologies.
5. Life-cycle assessment (LCA) is an essential instrument for assessing the environmental impact of materials, providing a detailed analysis of the ecological effects of FTW and FTB throughout their lifecycle, encompassing raw material extraction, production, usage, and disposal. Future investigations should utilize LCA to establish a scientific basis for these materials’ eco-friendly design and sustainable advancement. This methodology reduces the environmental impact during production and promotes the wider utilization of FTW and FTB across diverse sectors. By comprehensively evaluating their environmental consequences, sustainable production and development objectives for these materials can be more effectively achieved.

Despite various challenges, FTW and FTB, as emerging sustainable materials, are set to optimize the remarkable potential of bio-based materials through functional modifications. These materials have already been utilized in optoelectronic and sensor applications. With continuous improvements in their preparation methods and research on functional modifications, we expect that in the forthcoming years, flexible transparent wood, bamboo, and their derived functional materials will yield significant advancements in diverse areas, including smart buildings, energy conservation, energy conversion, sensors, and electronic skin.

## Figures and Tables

**Table 1:** Comparison of manufacturing methods and properties of FTW and FTB with biomass-derived materials.

Sample	Reagent Used	Polymer	Functional Additive	Optical Property		TS (MPa)	Ref.
				Tr (%)	Haze (%)		
TWF	NaClO	/	/	90%	80%	150 MPa	[96]
TWF	NaClO_2_/Acetate	EP	/	82%	/	75.12 MPa	[78]
TWF	Lignin modification	PVA	/	80%	90%	13.3 MPa	[89]
TWF	DES	PAA	CDs	85%	85%	60.92 MPa	[94]
TBF	NaClO_2_/Acetate	EP	/	81.47%	72.43%	169.03 MPa	[100]
TBF	NaClO_2_/Acetate	/	/	70.2%	88.1%	656.6 MPa	[99]
TBF	NaClO_2_/Acetate	EP	/	80%	72%	78.5 MPa	[90]
CNF	NaCIO	/	/	90%	/	350 MPa	[101]
CNF	cellulose nanofiber suspension oxidized	/	SiNPs	Tr:		/	[102]
				80% (0.1 wt% CNF)			
				30% (0.5 wt% CNF)			
BCF	BC nanofiber suspensions	PVA	/	Tr:			[103]
				90% (2.5 mL BC)		32.5 MPa (2.5 mL BC)	
				85% (7.5 mL BC)		37.5 MPa (7.5 mL BC)	
SPF	Silk Fibroin Solution	/	Yb^3+^/Er^3+^:YAG	/	/	7.8 MPa	[104]

Note: Transmittance (Tr); Tensile strength (TS); Transparent wood film (TWF); Transparent bamboo film (TBF); Cellulose nanofibers (CNF); Bacterial cellulose films (BCF); Silk protein films (SPF); Polyacrylic acid (PAA).

## Data Availability

Not applicable.
